# Efficacy and safety of intraarticular corticosteroid injections in adolescents with juvenile idiopathic arthritis in the temporomandibular joint: a Norwegian 2-year prospective multicenter pilot study

**DOI:** 10.1186/s12969-020-00464-3

**Published:** 2020-10-01

**Authors:** Paula Frid, Thomas A. Augdal, Tore A. Larheim, Josefine Halbig, Veronika Rypdal, Nils Thomas Songstad, Annika Rosén, Karin B. Tylleskär, Johanna Rykke Berstad, Berit Flatø, Peter Stoustrup, Karen Rosendahl, Eva Kirkhus, Ellen Nordal

**Affiliations:** 1 Department of Otorhinolaryngology, Division of Oral and Maxillofacial Surgery, University Hospital North Norway, Tromsø, Norway; 2 Public Dental Service Competence Centre of North Norway, Tromsø, Norway; 3 Pediatric Research Group, Department of Clinical Medicine, UiT the Arctic University of Norway, Tromsø, Norway; 4 Section for Pediatric Radiology, Department of Radiology, University Hospital of North Norway, Tromsø, Norway; 5 Department of Clinical Dentistry, UiT The Arctic University of Norway, Tromsø, Norway; 6 Department of Pediatrics and Adolescence Medicine, University Hospital of North Norway, Tromsø, Norway; 7 Department of Clinical Dentistry, University of Bergen, Bergen, Norway; 8 Department of Oral and Maxillofacial Surgery, Haukeland University Hospital, Bergen, Norway; 9 Department of Pediatrics, Haukeland University Hospital, Bergen, Norway; 10 Department of ENT and Oral and Maxillofacial Surgery, Oslo University Hospital, Oslo, Norway; 11 Department of Rheumatology, Oslo University Hospital, Oslo, Norway; 12 University of Oslo, Oslo, Norway; 13 Section of Orthodontics, Department of Dentistry and Oral Health, Aarhus University, Aarhus, Denmark; 14 Department of Radiology, Oslo University Hospital, Oslo, Norway

**Keywords:** Juvenile idiopathic arthritis, Temporomandibular joint, Intraarticular corticosteroids, Temporomandibular arthritis, Magnetic resonance imaging, Efficacy, Adverse events

## Abstract

**Background:**

Intraarticular corticosteroids (IACs) have been used to treat temporomandibular joint (TMJ) arthritis. However, prospective clinical studies with magnetic resonance imaging (MRI) scoring are lacking. The aim of this study was to examine efficacy and safety of a single IAC in the TMJ in adolescents with juvenile idiopathic arthritis (JIA) in a clinical setting.

**Methods:**

In this Norwegian prospective multicenter pilot study 15 patients with JIA (mostly persistent oligoarthritis or RF negative polyarthritis categories) and a clinically and MRI-verified diagnosis of TMJ arthritis were treated with IACs and followed for 2 years. Demographics, systemic medication, general disease activity and outcome measures were recorded including a pain-index score and maximal incisal opening (MIO). Inflammation and bone damage scores were assessed, using two recently published MRI scoring systems with masked radiological evaluation.

**Results:**

Among the 15 patients, 13 received a single IAC (5 bilateral), and 2 repeated IACs once unilaterally. Thus, the total number of IACs was 22. Median age was 15 years and the majority had an age not thought of as critical regarding mandibular growth retardation due to steroid injection. During the 2-year observation period systemic medication with disease modifying antirheumatic drugs (DMARDs) including biologics was initiated or adjusted in 10/15 (67%) patients. At the 2-months study visit after injection we observed a minimal improvement in MIO from median 44 (1st, 3rd quartiles; 36, 48) mm to 45 (43, 47) mm, *p* = 0.045 and decreased MRI mean additive inflammatory score from 4.4 ± 1.8 standard deviations (SD) to 3.4 ± 2.0, *p* = 0.040. From baseline to the 2-months follow-up pain improved in 6/11 patients but pain scores were not significantly improved. MRI-assessed damage increased in two patients with repeated IACs, and decreased in 3 patients but most of the patients were stable over the 2-year follow-up. Intra-rater repeatability of the MRI scoring system domains varied from poor to excellent.

**Conclusions:**

In this pilot study of predominately single IACs to the TMJ in combination with systemic treatment we observed improvement in MRI-assessed inflammation, mostly stable condylar bone conditions and minimal clinical improvement in adolescents with JIA and TMJ arthritis. No severe side effects were seen.

## Background

The temporomandibular joint (TMJ) is one of the most commonly involved joints in children with juvenile idiopathic arthritis (JIA), and may lead to impaired joint function, pain, growth impairment with dentofacial deformities [1–4], a reduced posterior airway space with related comorbidities [5, 6], and impaired quality of life [7]. The rate of TMJ arthritis varies significantly (40–90%) in different JIA-cohorts using magnetic resonance imaging (MRI) [8–10], as reviewed by Larheim et al. [11]. TMJ arthritis may be clinically silent with symptoms and signs seen only late in the disease course [3]. The diagnostic assessment is therefore a particular challenge. The definition of JIA [12] frequently cannot be applied to the TMJ [11].

Both systemic and local treatments have been used in patients with TMJ arthritis [13–15]. Several observational studies report short-term effect of intraarticular corticosteroid injections (IACs) to the TMJ on pain and maximal incisal opening (MIO) [16–21]. It has also been shown that the IACs can be safely performed by trained specialists both with the “landmark guiding technique” guided by anatomical landmarks, and with MRI, ultrasound or computed tomography (CT) guidance [17, 19, 22, 23]. However, treatment with IACs has been suspected to inflict rather than improve mandibular growth impairment [24, 25]. IACs to the TMJ may be performed with or without lavage for example by the Alstergren push-and-pull technique [26].

Retrospective studies with observation periods ranging from mean 2 to 23 months show a highly variable rate of improvement in MRI-assessed inflammation (18–83%) in TMJs receiving IACs, most often with stable condylar status on MRI [16, 17, 27–29]. However, according to Stoustrup et al. [30] and Stoll et al. [31] studies on IACs have a low level of evidence due to methodological issues. The studies are mostly retrospective and single center case-series, and the outcome assessors are not masked regarding pre- or post-treatment assessments. Randomized controlled trials are lacking, and systematic prospective follow-up studies with validated clinical assessments tools and imaging scoring systems are also missing. To our knowledge, there are no prospective studies with masked standardized MRI assessment addressing safety and efficacy of IACs in the TMJ in JIA. Therefore, the aim of this Norwegian 2-year prospective multicenter pilot study of adolescents with JIA and TMJ arthritis was, by using validated clinical outcome measures and two newly published MRI scoring systems, to assess efficacy and safety of single IACs in the TMJ in terms of (i) reducing pain and improving maximal mouth opening capacity, and (ii) reducing joint inflammation and bone damage.

## Material and methods

### Study design and patients

This 2-year prospective multicenter-study of IACs in adolescents with JIA and TMJ arthritis, is part of a larger Norwegian prospective multicenter cohort on JIA (www.norjia.com). The terminology adheres to TMJaw (earlier EuroTMJoint) consensus-based standardized terminology [32]. Clinical and demographic data were collected between November 2015 and September 2019 at the Department of Pediatrics, University Hospital North Norway Tromsø, Public Dental Service Competence Centre of North Norway, Tromsø, Haukeland University Hospital Bergen, and Oslo University Hospital, Rikshospitalet, Oslo.

Fifteen adolescents with JIA were consecutively recruited and a total of 22 TMJ injections with corticosteroids were performed. No control group was included due to ethical reasons. TMJ arthritis was defined as “clinical signs of pain on jaw movement, limitation of MIO, limitation of laterotrusive- or protrusive jaw movements or dentofacial growth disturbances and MRI signs of TMJ-arthritis (i.e. active inflammation in the TMJ based on increased contrast enhancement, bone marrow edema and/or effusion)”. Inclusion criteria were patients fulfilling the JIA diagnosis according to the classification criteria defined by the International League of Associations for Rheumatology (ILAR), age < 18 years, and arthritis in one or both TMJs. Exclusion criteria were contraindications to MRI such as cardiac pacemaker, metallic clips, contrast allergy etc. or previous TMJ IACs within the last 3 years. Non-invasive management in terms of physiotherapy and splint devices were considered and used by most adolescents in the study without sufficient effect before the decision to perform IACs was taken.

The included patients had a clinical examination and a MRI at baseline of the study before the TMJ IACs were performed, and at follow-up visits after 1–3 months, 1 year and 2 years.

### Clinical variables, TMJ examination, and assessment of disease activity

Demographics, systemic medication, JIA category, disease onset and course type of JIA, medication, and a general clinical examination including number of joints with active arthritis, were registered by specialists in pediatric rheumatology at each study visit. The physician global evaluation of overall disease activity on a 10-cm visual analogue scale (VAS) (MDgloVAS) was also assessed at the visit. The specialists were calibrated by thorough discussions of all study variables assessed in the NorJIA study and the present NorJIA TMJ injection substudy. Patient-reported global assessment of overall well-being (PRgloVAS) and patient-reported pain (PRpainVAS) within the last week on a 10-cm VAS were also collected. On these scales, 0 indicates no disease activity/no pain/best overall well-being, and 10 indicate the maximum disease activity/worst pain/poorest overall well-being, respectively [33]. Number of active joints other than the TMJ was defined according to the clinical definition of arthritis: swelling within a joint or limitation in the range of joint movement with joint pain or tenderness [34].

Clinical TMJ examination was performed by either a specialist in oral and maxillofacial surgery or a specialist in pediatric dentistry (PF, AR, JRB, JH) according to the DC / TMD examination and diagnostics protocol [35] and EuroTMJoint Clinical Recommendations protocol [36]. The two examiners were calibrated repeatedly during the study period [37]. The TMJ clinical outcomes for this study were: 1, Pain-index score (i.e. pain frequency last 2 weeks x pain intensity last 2 weeks (VAS 0–10)) scored by the patient, 2, Maximal incisal opening (MIO) in mm scored by the clinical TMJ examiner and 3, jaw function the last 2 weeks (VAS 0–10) scored by the patient was registered.

A routine complete blood cell count, erythrocyte sedimentation rate (ESR) (mm/hour), and C-reactive protein (CRP) (< 5 mg/l was set as 0) was obtained. Rheumatoid factor (RF) immunoglobulin M was analyzed twice more than 3 months apart. The composite juvenile arthritis disease activity score (JADAS10, range from 0 to 40) was assessed, based on the MDgloVAS (range 0–10), PRgloVAS (range 0–10), active joint count (maximum 10 joints), and the ESR (normalized to 0–10, < 10 mm/h was set as 0) [33, 38]. Disease status was defined as either active disease, inactive disease, clinical remission on medication, or clinical remission off medication according to the ACR provisional criteria [39]; inactive disease requiring all the following: 1, no active joints; 2, no fever, rash, serositis, splenomegaly or generalized lymphadenopathy attributable to JIA; 3, no active uveitis; 4, normal ESR or CRP; 5, MDgloVAS =0; and 6, duration of morning stiffness of ≤15 min. Side effects were assessed and registered as per protocol at each study visit, including any signs of infection, bleeding, skin atrophy, facial palsy, or intraarticular calcifications on MRI.

### MRI method and outcomes

Fifty-seven examinations were obtained on either 3-T-units (Magnetom Skyra, Siemens Healthcare, Erlangen, Germany) using a 64-channel head/neck coil (*n* = 50) or a 1.5-T-unit (Magnetom Aera or Avanto, Siemens Healthcare, Erlangen, Germany) using 4-channel special purpose coils (*n* = 7), according to protocol A or B, respectively. As a minimum the protocols included water-sensitive images, pre- and post-contrast T1-weighted images and one sequence with the mouth in the open position. Details are provided in Supplementary Table 1. One examination had susceptibility artefact-reducing sequences (WARP). The contrast medium given was 0.2 mL/kg (0.1 mmol/kg) body weight gadoterate meglumine (Dotarem, Guerbet, Paris, France). None of the patients needed sedation. All examinations were reviewed on a PACS workstation (IDS7, Sectra Medical Systems, Linköping, Sweden) in consensus between two experienced specialists in radiology (TAL, TAA) at random order, masked for personal data, injection laterality and time point. One examination from each subject was randomly selected for a second reading after an interval of approximately 3 months, to assess intra-rater variability. Inter-rater variability was not assessed in this study.

The image outcomes were based on inflammation and bone damage according to the two newly published MRI scoring systems for evaluating TMJ arthritis as described by Tolend et al. [40, 41] and Kellenberger and Lochbuhler et al. [25, 42]. The scoring systems were thoroughly discussed between the radiologists before the reading session. Total scores ranged from 0 to 8 for the Additive inflammatory domain (bone marrow edema (absent/present 0/1), bone marrow enhancement (absent/present 0/1), joint effusion (absent/small/large 0/1/2), synovial thickening (absent/mild/moderate, severe 0/1/2), joint enhancement (absent/mild/moderate, severe 0/1/2)) and 0–5 in the Additive damage domain (condylar flattening (absent/mild/moderate, severe 0/1/2), erosions (absent/mild/moderate, severe 0/1/2), disc abnormalities (absent/present 0/1)), and 0–4 in the Progressive scoring system: Progressive inflammation (no inflammation/mild/ moderate/severe/pannus 0/1/2/3/4) and Progressive osseous deformity (normal/mild/moderate/ severe/destruction 0/1/2/3/4). The scores were set as missing if the images were not of sufficient quality due to braces or other artefacts. In case of bilateral injection, the joint with the most severe inflammation/bone damage was chosen for statistical analysis.

### Injection procedure

The preauricular skin was disinfected with 70% ethanol and 5% chlorhexidine, before local anesthesia with an auriculotemporal nerve block was applied. The push-and-pull technique, and the amount of recovered synovial fluid in each sample was quantified with the hydroxocobalamin method, as described by Alstergren et al. [26, 43]. A washing solution consisting of 22% hydroxocobalamin (Behepan® 1 mg/ml) in physiological saline (sodium chloride 9 mg/ml) was used. The TMJ was injected with a total of 4 ml washing solution through a stop-cock syringe. One milliliter of washing solution was injected slowly, the valve was turned and then as much fluid as possible was aspirated back. This procedure was repeated a total of three times for each joint leaving the same cannula inside the joint. If aspiration of the washing solution was possible and the resistance in the syringe was minor during injection, then the needle tip was considered to be placed within the joint space. After sampling of synovial fluid from the upper joint compartment, steroids were injected according to the landmark guiding technique, 1 cm anterior and 2 mm inferior to the tragus of the ear. Two different types of steroids were used: methylprednisolone acetate (Depomedrol®) and triamcinolone hexacetonide (Lederspan®). Methylprednisolone acetate was used in one of the first patients because this was used by Alstergren et al. in their studies on the push-and-pull technique to the TMJ. Shortly after start of the study we agreed to use solely triamcinolone hexacetenoide [26]. The following dosages were set: patients > 30 kg: 0.4 ml triamcinolone hexacetonide 20 mg/ml and children < 30 kg: individual dosage. In 15 TMJs a syringe of 25G 0.5 × 25 mm and in 7 TMJs a syringe of 23G 0.6 × 30 mm was used for the injection. The injection procedure was performed by experienced specialists in oral and maxillofacial surgery at all centers (PF, AR, JRB). The results of the synovial fluid analyses will be published in a separate paper.

### Statistical analyses

For description of clinical and demographic data, median (1st, 3rd quartiles), mean (standard deviations (SD)) and frequencies (percentage) were used as appropriate. For the not normally distributed data of MRI-scoring mean (SD) was used for more informative description of the values. Multiple testing of four time-points and Bonferroni correction for 6 comparisons with a *p*-value < 0.008 was analyzed and considered, but found to be less informative than testing for two time-points; with differences between baseline and 2-months, and 2-year follow-up because of some missing data at different follow-up time-points, varying number of follow-up visits from 2 to 4, together with the low number of participants. Based on previous studies [16, 17, 27–29] and clinical experience we chose to assess change in clinical parameters (MIO and pain index) and change in the MRI inflammatory scores mainly between baseline and 2-months, while change in MRI osseous deformities and damage scores was assessed between baseline and 2-year follow-up.

**Fig. 1:**
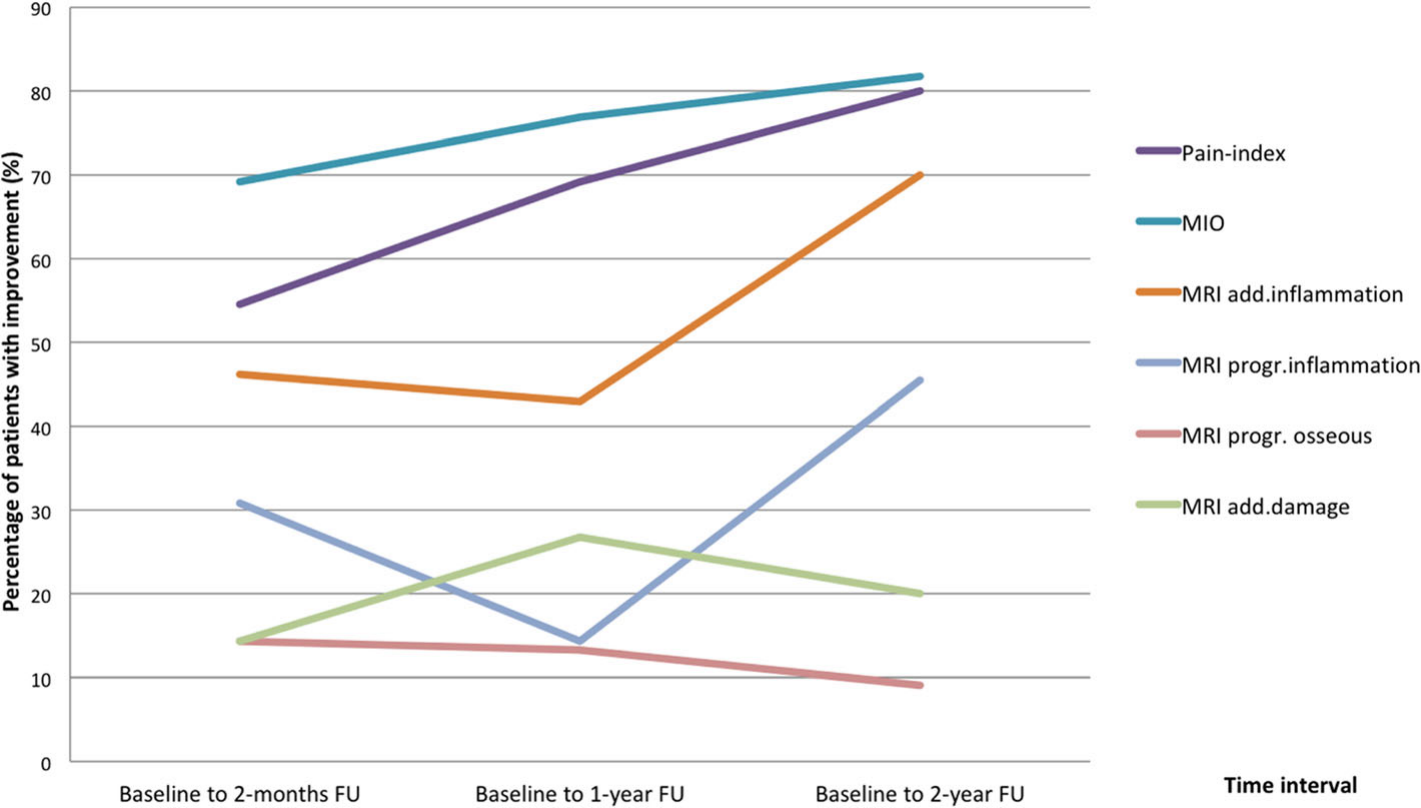
Percentage of patients with improvement. Percentage of adolescents with juvenile idiopathic arthritis (JIA) and temporomandibular joint (TMJ) arthritis with improvement in Pain index, maximal incisal opening (MIO) and magnetic resonance imaging (MRI) inflammatory additive domain score, damage additive domain score, progressive inflammation score, progressive osseous deformity score, in the time interval between baseline and follow-up visits (FU) after receiving intraarticular corticosteroids (IACs)

When testing continuous data for differences between baseline and 2-months, and 2-year follow-up, the Wilcoxon signed-rank test was used for not normally distributed data. For nominal data tested for differences between baseline and 2-months, and 2-year follow-up McNemar Chi-square test was used, and for ordinal data Wilcoxon signed-rank test. A *p*-value < 0.05 was considered statistically significant. For description of outcome after receiving IACs at the different time-points in Fig. 1, percentage of patients were used for absolute improvement of the variables pain, MIO and MRI. For the MRI assessment, the intra-observer consensus agreement for the MRI-scoring was assessed with Cohen’s kappa: poor (0.01–0.20), fair (0.21–0.40), moderate (0.41–0.60), substantial (0.61–0.80) and almost perfect (0.81–1.00) agreement. Statistical analysis was performed using SPSS software, versions 25 or 26.

## Results

### Demographic and disease activity parameters

Demographic and disease activity characteristics at baseline are given in Table 1. In total 15 adolescents were included and 22 TMJ injections were performed in this study. Among the 15, 80% were female and the median (quartiles) age at baseline was 15 (1st, 3rd quartiles 11, 16) years. The majority of adolescents belonged to the persistent oligoarthritis (6/15; 40%) or the RF negative polyarthritis (5/15; 33%) JIA categories. Five patients (33%) received bilateral TMJ IACs. Two patients (13%) had repeated injections once unilaterally 11 and 13 months after baseline, on indication pain and ongoing MRI-assessed inflammation. Ten of 22 TMJs were sampled with the push-and-pull method (46%). Follow-up visits were performed at a median of 2.0 (1.8, 3.3) months (*n* = 14), 12.0 (11.0, 13.0) months (*n* = 15), and 22.0 (22.0, 23.0) months (*n* = 11) after TMJ injections at baseline. All patients had active disease at baseline. At the 2-year follow-up five of 11 (46%) patients were in remission either on or off medication. During the 2-year follow-up period 10/15 (67%) changed or increased their systemic medication with DMARDs and biologics. From participant centers, two patients were included in Oslo, one patient in Bergen and 12 patients in Tromsø.

**Table 1:** Characteristics at baseline in adolescents with JIA (*n* = 15) receiving IACs to the TMJs (*n* = 22)

Baseline characteristic	Value
Female, no. (%)	12 (80)
Age at injections, yrs	15 (11, 16)
Age at JIA onset, yrs.	11 (8, 14)
Disease duration, yrs.	1 (0, 5)
**JIA-category, no (%)**	
Oligoarthritis persistent	6 (40)
Polyarthritis RF negative	5 (33)
Oligoarthritis extended	3 (20)
Enthesitis related arthritis	1 (7)
**Disease activity, no (%)**^a^	
Active	15 (100)
Remission on medication	–
Remission off medication	–
**Medication baseline, no (%)**	
No DMARDs	6 (40)
DMARDs (MTX)	3 (20)
Biologics combination	6 (40)
**Disease activity variables**	
JADAS10 baseline (*n* = 8)	15.8 (12.9, 49.1)
No.of active joints (*n* = 14)	2.0 (1.0, 3.0)
PRpainVAS (*n* = 10)	4.8 (3.3, 7.6)
PRgloVAS (*n* = 10)	5.5 (3.3, 7.1)
MDgloVAS (*n* = 12)	2.5 (1.6, 4.5)
ESR (mm/h) (*n* = 12)	6.5 (3.5, 10.5)
TMJ-examination to injection, days	14.0 (1.0, 68.0)
Follow-up, months	22.0 (16.0, 23.0)
Injection to 2-months follow-up, (*n* = 14)	2.0 (1.8, 3.3)
Injection to 1-year follow-up, months (*n* = 15)	12.0 (11.0, 13.0)
Injection to 2-year follow-up, months (*n* = 11)	22.0 (22.0, 23.0)
Triamcinolone hexacetonide, dose, mg (*n* = 14)	20.0 (9.5, 20.0)
Methylprednisolone acetate, dose, mg (*n* = 1)	40.0 (*n* = 1)
Push-pull technique / No. TMJs (%)	10/22 (46)
Needle length, mm/ No. TMJs	25 mm/15, 30 mm/7

Data are median (1st, 3rd quartile) unless otherwise indicated. Two patients received repeated injection on the same side, five patients received bilateral injection

*IACs* intraarticular corticosteroid injection, *JIA* juvenile idiopathic arthritis, *TMJ* temporomandibular joint, *PRpainVAS* patient reported pain visual analogue scale, *PRgloVAS* patient reported global assessment of well-being, *MDgloVAS* medical doctor global assessment of well-being, *JADAS10* the composite juvenile arthritis10-joint disease activity score, *ESR* erythrocyte sedimentation rate, *MTX* methotrexate, *DMARDs* disease modifying antirheumatic drugs

^a^disease activity status according to the ACR provisional remission criteria [39]

### Clinical outcomes

Among the clinical TMJ parameters pain-index score changed from median 6.0 (0.0–13.0) at baseline to 2.0 (0.0–10.0) at 2-months follow-up, this was not a statistically significant improvement (*p* = 0.263). There was a minimal, but statistically significant increase in MIO during the same observation period (*p* = 0.045) (Table 2). At 2-year follow-up, scores for pain and jaw function improved from baseline in terms of pain frequency (*p* = 0.016), pain intensity (*p* = 0.012), VAS jaw function (*p* = 0.034), and pain-index score (*p* = 0.012) (Table 2).

**Table 2:** Disease activity and TMJ clinical measures during 2-year follow-up in 15 adolescents with JIA and TMJ-arthritis receiving IACs

	Pre- injection (T0)	2-months FU (T1)	1-year FU (T2)	2-year FU (T3)	*p*-value T0-T1
Median months after IACs	0	2.0 (1.8, 3.3)	12.0 (11.0, 13.0)	22.0 (22.0, 23.0)	
(1st, 3rd quartile)	*n* = 15	*n* = 14	*n* = 15	*n* = 11	
Disease activity, no (%)**					
Active	15 (100)	7 (54) *n* = 13	10 (77) *n* = 13	6 (55)	
Remission on medication	–	2 (15) *n* = 13	2 (15) *n* = 13	2 (18)	
Remission off medication	–	4 (31) *n* = 13	1 (8) *n* = 13	3 (27)	
Medication ***					
No DMARDs, no (%)	6 (40)	5 (39) *n* = 13	4 (27)	3 (27)	0.317 ^a^
DMARDs (MTX), no (%)	3 (20)	2 (15) *n* = 13	3 (20)	3 (27)	
Biologics comb, no (%)	6 (40)	6 (46) *n* = 13	8 (53)	5 (46)	
**Disease activity measures**					
JADAS10	15.8 (12.9, 49.1) *n* = 8	11.0 (6.0, 20.0) *n* = 7	12.5 (6.8, 14.5) *n* = 9	8.5 (3.3, 15.3) *n* = 4	0.273^b^
No.of active joints	2.0 (1.0, 3.0) *n* = 14	0.0 (0.0, 2.0) *n* = 13	1.0 (0.0, 1.5) *n* = 13	0.0 (0.0, 1.0) *n* = 10	0.076^b^
ESR (mm/h)	6.5 (3.5, 10.5) *n* = 12	6.0 (3.8, 11.0) *n* = 10	5.0 (3.0, 7.0) *n* = 11	5.5 (3.8, 7.3) *n* = 10	0.445^b^
PRpainVAS	4.8 (3.3, 7.6) *n* = 10	3.5 (0.0, 5.8) *n* = 8	3.8 (1.6, 6.3) *n* = 10	1.5 (0.0, 4.5) *n* = 4	0.500^b^
PRgloVAS	5.5 (3.3, 7.1) *n* = 10	3.5 (0.1, 5.8) *n* = 8	4.0 (1.6, 5.1) *n* = 10	0.5 (0.0, 4.0) *n* = 4	0.345^b^
MDgloVAS	2.5 (1.6, 4.5) *n* = 12	0.5 (0.0, 4.0) *n* = 10	1.5 (0.1, 2.8) *n* = 12	0.0 (0.0, 1.0) *n* = 7	0.207^b^
**TMJ activity measures**					
Pain frequency	2.0 (0.0, 4.0)	1.0 (0.0, 2.0) *n* = 11	1.0 (0.0, 2.0) *n* = 13	0.0 (0.0, 2.0) *n* = 10	0.245^b^
VAS pain intensity	3.0 (0.0, 6.5) *n* = 15	2.0 (0.0, 4.5) *n* = 11	2.0 (0.0, 3.5) *n* = 13	0.0 (0.0, 2.1) *n* = 10	0.292^b^
VAS jaw function	3.0 (0.0, 4.3) *n* = 13	0.0 (0.0, 2.4) *n* = 10	0.0 (0.0, 0.0) *n* = 13	0.0 (0.0, 2.8) *n* = 9	0.201^b^
Pain index**γ**	6.0 (0.0, 13.0) *n* = 15	2.0 (0.0, 10.0) *n* = 11	2.0 (0.0, 8.5) *n* = 13	0.0 (0.0, 5.3) *n* = 10	0.263^b^
MIO (mm)	44 (36, 48) *n* = 15	45 (43, 47) *n* = 13	45 (42, 49) *n* = 13	46 (45, 48) *n* = 11	0.045^b^

Data are median (1st, 3rd quartiles) unless indicated otherwise. ^a^McNemar chi square test, ^b^Wilcoxon signed-rank test, *p ≤ 0.05 for statistical significance ** remission, status according to the ACR provisional remission criteria [39]***No DMARDs, Current us of NSAIDs and/or IACs; DMARDs, current use alone of MTX; Biologics comb, current use of Biologics alone or in combination with MTX; **γ** Pain index = Pain frequency last 2 weeks x Pain intensity last 2 weeks (VAS 0–10); *JIA* juvenile idiopathic arthritis, *TMJ* temporo-mandibular joint, *LOM* limited range on motion, *VAS* visual analogue scale, *MTX* methotrexate, *DMARDs* disease modifying antirheumatic drugs, *PRpainVAS* patient reported pain visual analogue scale, *PRgloVAS* Patient reported global assessment of well-being, *MDgloVAS* medical doctor global assessment of well-being, *JADAS10* The composite juvenile arthritis 10-joint disease activity score, *IACs* intraarticular corticosteroid injections, *ESR* erythrocyte sedimentation rate, *MIO* maximal incisal opening, *FU* follow-up

Absolute improvement in pain-index was seen in 6/11 (55%) of the patients at 2-months follow-up, 9/13 (69%) of the patients at 1-year follow-up and 8/10 (80%) of the patients at 2-year follow-up (Fig. 1). Two of 11 patients (18%) had increased pain and 3 (27%) unchanged pain from baseline to 2-months follow-up. At 2-year follow-up 2 of 10 patients (20%) had a stable pain-index score of zero, and none of the patients had increased pain-index compared to baseline. Absolute improvements in MIO at the three follow-up visits were 9 of 13 (69%), 10 of 13 (77%) and 9 of 11 (82%) respectively (Fig. 1). In 3 of 13 patients (23%) MIO decreased at 2-months follow-up (48 to 42 mm, 46 to 45 mm, 45 to 44 mm respectively). In 2 of 11 patients (18%) MIO decreased (40 to 37 mm, 49 to 45 mm respectively) between baseline and 2-year follow-up. Improvements in MIO ≥5 mm at the three follow-up visits were 4 of 13 (31%), 6 of 13 (46%) and 5 of 11 (46%) respectively.

### MRI outcomes

**Fig. 2:**
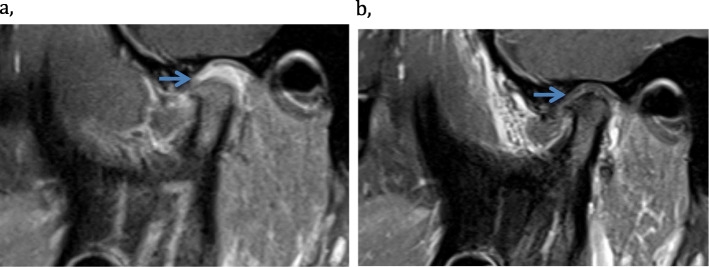
MRI improvement of the inflammation. Oblique sagittal contrast enhanced T1 TSE images with fat suppression of a 16-year-old girl (case 8) (**a**) at baseline with increased temporomandibular joint enhancement (blue arrow) and (**b**) at 2 months follow-up after IAC and no DMARDs with complete regression of joint enhancement (blue arrow). Note also the disrupted disc and flattened condyle in both images

There was a statistically significant reduction in the additive inflammatory domain from baseline to the 2-months- and from baseline to the 2-year follow-up, (*p* = 0.040, *p* = 0.017 respectively) (Table 3) (Fig. 1). At the 2-months follow-up, 6 of 13 patients (46%) had lower score as shown in Fig. 1, while 6 (46%) had unchanged and 1 (8%) had higher score in the additive inflammatory score. Among the 10 patients at 2-year follow-up, 7 (70%) had lower score, while 3 (30%) remained unchanged. The MRIs at baseline and at follow-up 2 months after IAC in one of the patients with improvement of temporomandibular joint enhancement is shown in Fig. 2.

**Table 3:** Additive and progressive scoring system for assessment of inflammation and damage in the temporomandibular joint (TMJ) by magnetic resonance imaging (MRI) in 15 adolescents with juvenile idiopathic arthritis (JIA) and TMJ-arthritis receiving intraarticular corticosteroids (IACs)

	Pre- injection (T0)	2- months FU (T1)	1-year FU (T2)	2-year FU (T3)	*p*-value
Mean months after IACs (SD)	0 *n* = 15	2.4 ± 1.6 *n* = 14	12.3 ± 1.5 *n* = 15	21.5 ± 2.6 *n* = 11	
**Additive Inflammatory domain:** (Bone marrow edema, bone marrow enhancement, Joint effusion, Synovial thickening, Joint enhancement)	4.4 ± 1.8	3.4 ± 2.0 *n* = 13	3.6 ± 1.7 *n* = 14	2.3 ± 1.7 *n* = 10	0.040^* a^
Bone marrow edema	0.3 ± 0.5	0.1 ± 0.3	0.1 ± 0.4 *n* = 14	0.0 ± 0.0 *n* = 10	0.500^* b^
Bone marrow enhancement	0.4 ± 0.5	0.2 ± 0.4 *n* = 13	0.1 ± 0.4 *n* = 14	0.0 ± 0.0 *n* = 11	0.250^* b^
Joint effusion	0.8 ± 0.9	0.7 ± 0.8 *n* = 14	0.6 ± 0.9 *n* = 14	0.3 ± 0.7 *n* = 10	0.705^* a^
Synovial thickening	1.0 ± 0.8	1.0 ± 0.8 *n* = 14	1.0 ± 0.8 *n* = 14	0.7 ± 0.8 *n* = 10	1.000^* a^
Joint enhancement	1.9 ± 0.3	1.5 ± 0.7 *n* = 13	1.8 ± 0.4 *n* = 14	1.3 ± 0.6 *n* = 11	0.059^* a^
**Additive Damage domain:** (Condylar flattening, erosions, disc abnormalities)	2.6 ± 1.5	2.8 ± 1.5 *n* = 14	2.7 ± 1.6	2.5 ± 1.7 *n* = 10	1.000^** a^
Condylar flattening	1.3 ± 0.9	1.6 ± 0.8 *n* = 14	1.5 ± 0.9	1.5 ± 0.9 *n* = 11	0.157^** a^
Erosions	0.6 ± 0.7	0.6 ± 0.8 *n* = 14	0.5 ± 0.8	0.4 ± 0.7 *n* = 10	0.655^** a^
Disc abnormalities	0.7 ± 0.5	0.6 ± 0.5 *n* = 14	0.7 ± 0.5	0.7 ± 0.5 *n* = 11	1.000^** b^
**Progressive inflammation**	2.6 ± 0.8	2.0 ± 1.1 *n* = 13	2.5 ± 1.0 *n* = 14	1.5 ± 0.9 *n* = 11	0.066^* a^
**Progressive osseous deformity**	2.0 ± 1.3	2.1 ± 1.1 *n* = 14	2.0 ± 1.4	2.0 ± 1.3 *n* = 11	1.000^** a^

Values are the mean ± SD unless indicated otherwise. *N* = 15 unless indicated otherwise. ^a^Wilcoxon signed-rank test. ^b^McNemar chi square test. ^*^P ≤ 0.05 considered statistically significant between T0-T1 and ^**^ between T0-T3. Each joint is scored independently (the worst joint is chosen when bilateral injection), with possible total scores ranging from 0 to 8 in the Additive inflammatory scoring system and 0–5 in the Additative damage domain, and 0–4 in the Progressive scoring system according to (Tolend et al.) [40] and (Kellenberger et al.) [41]; 2 patients received repeated injection on the same side, 5 patients received bilateral injection

There was no statistically significant change in the mean progressive inflammation score between baseline and 2-months follow-up; 4 of 13 (31%) had lower score, and 9 of 13 (46%) had unchanged score. At the 2-year follow-up; 5 of 11 patients (46%) had lower score and 6 (55%) was unchanged compared to baseline.

**Fig. 3:**
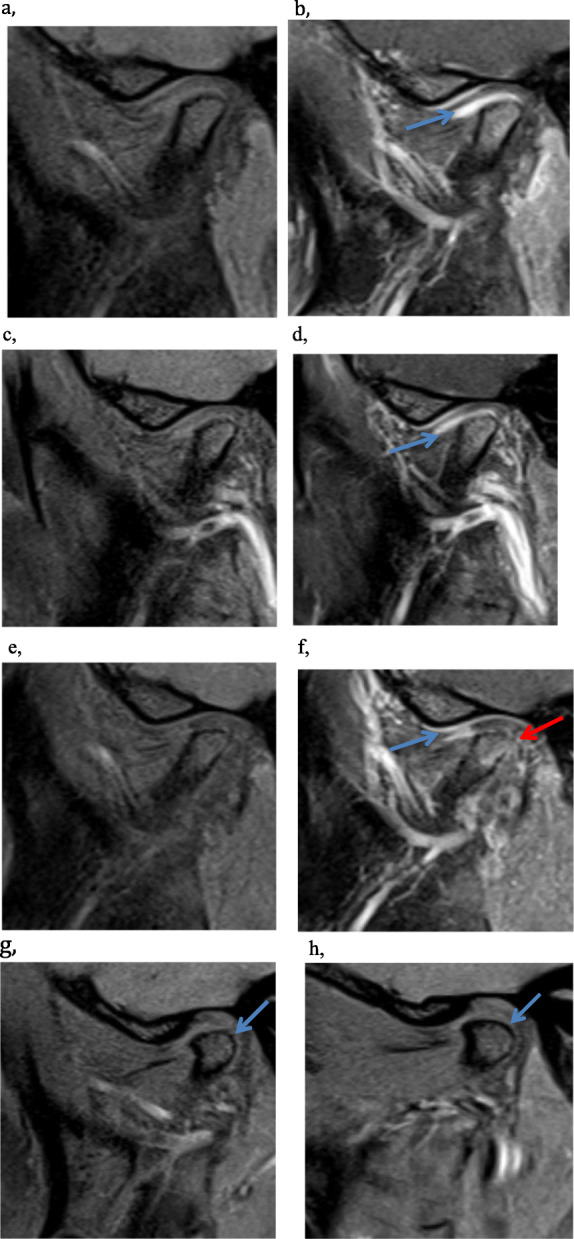
MRI changes of the bone condition. Oblique sagittal pre- and postcontrast T1 TSE images with fat suppression of the left TMJ of a 16-year-old girl (case 10) without improvement in inflammation: joint enhancement is only minimally reduced from baseline (**a**, **b**) to 2-months after IAC (**c**, **d**). She was under MTX treatment. At 2-year follow up, with a repeated injection 11 months after baseline, there is some reduction in joint enhancement (blue arrow), but the disc has become perforated and the condylar surface discretely more flattened and irregular (red arrow) (**e**, **f**). Oblique sagittal T1 TSE images with fat suppression of the right TMJ of a 15-year-old girl (case 9) with improved bone condition: discretely flattened and irregular condyle (blue arrow) at baseline (**g**) has become smooth and more rounded (blue arrow) at 2-year follow-up after IAC and systemic treatment with biologics and MTX (**h**)

No statistically significant change was seen in the two bone damage scores. In the mean additive damage domain, 2 of 14 (14%) had lower score, 9 (64%) unchanged and 3 (21%) had increased score at 2-months follow-up. At 2-year follow-up 2 of 10 (20%) had lower score, 6 (60%) was unchanged and 2 (20%) had higher score. The MRIs at baseline and at follow-up 2 years after IAC and systemic treatment show aggravation of bone damage in one patient (Case 10) and improvement in case 9 as shown in Fig. 3. In the mean progressive osseous deformity score at the 2-months follow-up, 2 of 14 (14%) had a lower score, 11 (79%) had unchanged score, and 1 (7%) had higher score. At the 2-year follow-up 1 of 11 (9%) had lower score, 9 (82%) was unchanged, 1 (9%) had higher score.

### MRI score intra-observer agreement

For the additive inflammatory domain, the intra-observer agreement between the readings was poor for bone marrow edema and bone marrow enhancement (negative kappa values), fair and moderate for joint effusion (kappa values 0.38 and 0.46), substantial and almost perfect for synovial thickening (0.63 and 0.90) and moderate and poor for joint enhancement (0.41 and 0.00) for the right and left side, respectively. The corresponding value for the progressive inflammation and progressive osseous deformity score was moderate and poor (0.49 and 0.20) and moderate (0.52 and 0.58). For the additive osseous domain, the intra-observer agreement was moderate to substantial for flattening (0.48 and 0.74), fair and moderate for erosions (0.36 and 0.44) and fair and almost perfect for disc abnormalities (0.36 and 0.84).

### Side effects

No severe adverse events that could be related to the IACs were reported, and there was no finding of intraarticular calcifications on MRI. One of the adolescents experienced pain after the injection but this minor side effect resolved within 1 month. Increased additive damage domain score was seen in 2/10 (20%) and 1/11 (9%) had increased progressive osseous deformity score in the TMJ between baseline and 2-year follow-up. Both patients had ongoing MRI-assessed inflammation at 2-year follow-up and repeated IACs 11 and 13 months respectively after baseline. Among these one had lower pain index and the other one unchanged (=0), and both patients had increased MIO at the 2-year follow-up. Furthermore, two patients with only 1-year follow-up (cases 1 and 5) had increased scores according to additive damage domain and progressive osseous deformity, together with ongoing inflammation (Table 4). Also, case 1 had a trauma to the mandible, a blow against one of the condyles, in between the 2-month and the 1-year follow-ups. One of the patients, case 7, improved according to the progressive osseous deformity from score 1 at the 2-months follow-up to score 0 at the 2-year follow-up. Mandibular growth was not evaluated because the adolescents in this study had mostly finished their growth at time for TMJ injection: median age 15.0 (11.0, 16.0) years.

**Table 4:** Summary characteristics, use of medication at baseline and outcome-response during 2-year follow-up in adolescents with (JIA) (*n* = 15) receiving (IACs) to the (TMJs) (*n* = 22)

Case	M/F	Age Onset	Age Inj	Dose inj (mg)	Medic (T0)	Medic (T1)	Medic (T2)	Medic (T3)	MIO	Pain index	MRI additive inflammatory domain^a^	MRI Progressive inflammation^a^	MRI additive damage domain^a^	MRI progressive osseous deformity^a^	Comments
1	M	10	10	6	MTX	MTX	MTX		T0: 48 T1: 42 T2: 48 T3: -	T0: 6 T1 T2: 2 T3: -	T0: 6 T1: 4 T2: 6 T3: -	T0: 3 T1: 3 T2: 4 T3: -	T0: 3 T1: 4 T2: 4 T3: -	T0: 3 T1: 3 T2: 4 T3: -	Bilateral IACs Mandibular trauma between T1 and T2
2	F	17	17	8	MTX	BioCo	BioCo	BioCo	T0: 40 T1: - T2: 37 T3: -	T0: 0 T1: - T2: - T3: -	T0: 3 T1: 2 T2: 5 T3: 1	T0: 3 T1: 2 T2: 3 T3: 1	T0: 4 T1: 4 T2: 3 T3: 3	T0: 3 T1: 3 T2: 3 T3: 3	Unilateral IACs
3	F	11	11	8	MTX	MTX	BioCo		T0: 32 T1: 35 T2: 30 T3: -	T0: 9 T1: 0 T2: 0 T3: -	T0: 5 T1: 5 T2: 5 T3: -	T0: 3 T1: 3 T2: 3 T3: -	T0: 3 T1: 3 T2: 2 T3: -	T0: 3 T1: 2 T2: 0 T3: -	Unilateral IACs
4	F	13	14	20	No DMARD	No DMARD	No DMARD	No DMARD	T0: 44 T1: 48 T2: 41 T3: 47	T0: 0 T1: 0 T2: 9 T3: 0	T0: 4 T1: 2 T2: - T3: - T4: -	T0: 4 T1: 1 T2: - T3: 1 T4: 2	T0: 2 T1: 4 T2: 5 T3: 4 T4: 4	T0: 2 T1: 2 T2: 3 T3: 3 T4: 3	Unilateral repeated IACs (13 months interval)
5	F	9	15	20	BioCo		BioCo		T0: 48 T1: - T2: - T3: -	T0: 3 T1: - T2: - T3: 0	T0: 6 T1: - T2: 6 T3: -	T0: 3 T1: - T2: 3 T3: -	T0: 1 T1: - T2: 4 T3: -	T0: 0 T1: - T2: 2 T3: -	Unilateral IACs
6	F	9	14	10	BioCo	BioCo	BioCo	BioCo	T0: 46 T1: 45 T2: 47 T3: 47	T0: 5.5 T1: 2 T2: 0 T3: 0	T0: 1 T1: 1 T2: 1 T3: 1	T0: 1 T1: 1 T2: 1 T3: 1	T0: 0 T1: 0 T2: 0 T3: 0	T0: 0 T1: 0 T2: 0 T3: 0	Bilateral IACs
7	F	2	15	20	No DMARD	No DMARD	No DMARD	MTX	T0: 36 T1: 44 T2: 44 T3: 44	T0: 36 T1: 10 T2: 6 T3: 0	T0: 3 T1: 3 T2: 3 T3: 1	T0: 1 T1: 1 T2: 2 T3: 1	T0: 1 T1: 2 T2: 1 T3: 1	T0: 0 T1: 1 T2: 0 T3: 0	Bilateral IACs
8	F	15	16	40^b^	No DMARD	No DMARD	MTX		T0: 45 T1: 46 T2: 50 T3: -	T0: 0 T1: 0 T2: 12 T3: -	T0: 2 T1: 2 T2: 2 T3: -	T0: 2 T1: 2 T2: 2 T3: -	T0: 4 T1: 4 T2: 4 T3: -	T0: 2 T1: 2 T2: 2 T3: -	Unilateral IACs
9	F	0	15	20	BioCo	BioCo	BioCo	No DMARD	T0: 49 T1: 54 T2: 54 T3: 45	T0: 0 T1: 5 T2: 0 T3: -	T0: 2 T1: 0 T2: 2 T3: 2	T0: 2 T1: 0 T2: 2 T3: 2	T0: 4 T1: 2 T2: 2 T3: 2	T0: 2 T1: 2 T2: 2 T3: 2	Unilateral IACs
10	F	15	16	20	No DMARD	No DMARD	MTX	MTX	T0: 45 T1: 44 T2: 49 T3: 48	T0: 12 T1: 22.5 T2: 0 T3: 6	T0: 5 T1: 6 T2: 4 T3: 2 T4: 3	T0: 3 T1: 3 T2: 3 T3: 1 T4: 3	T0: 3 T1: 3 T2: 3 T3: 4 T4: 3	T0: 3 T1: 3 T2: 3 T3: 3 T4: 3	Unilateral repeated IACs (11 months interval)
11	M	11	16	20	No DMARD	No DMARD	No DMARD	No DMARD	T0: 55 T1: 55 T2: 62 T3: 63	T0: 0 T1: 0 T2: 0 T3: 0	T0: 5 T1: 4 T2: 2 T3: 5	T0: 2 T1: 1 T2: 1 T3: 2	T0: 3 T1: 3 T2: 3 T3: 3	T0: 3 T1: 2 T2: 2 T3: 2	Unilateral injection
12	M	8	9	10	BioCo	BioCo	BioCo	BioCo	T0: 34 T1: 40 T2: 41 T3: 45	T0: 7.5 T1: 0 T2: 0 T3: 0	T0: 7 T1: 3 T2: 1 T3: 0	T0: 3 T1: 3 T2: 1 T3: 0	T0: 0 T1: 0 T2: 0 T3: 0	T0: 0 T1: 0 T2: 0 T3: 0	Bilateral IACs
13	F	14	15	16	BioCo	BioCo	BioCo	BioCo	T0: 42 T1: 44 T2: 45 T3: 46	T0: 21 T1: 14 T2: 2.5 T3: 5	T0: 6 T1: 6 T2: 5 T3: 5	T0: 3 T1: 3 T2: 3 T3: 3	T0: 5 T1: 5 T2: 5 T3: 5	T0: 3 T1: 3 T2: 3 T3: 3	Unilateral IACs
14	F	5	11	20	BioCo	BioCo	BioCo	BioCo	T0: 43 T1: 45 T2: 44 T3: 48	T0: 13 T1: 9 T2: 9 T3: 12	T0: 5 T1: - T2: 4 T3: 3	T0: 3 T1: - T2: 3 T3: 3	T0: 3 T1: 2 T2: 2 T3: -	T0: 3 T1: 3 T2: 3 T3: 3	Unilateral IACs
15	F	13	15	20	No DMARD	-	No DMARD	MTX	T0: 35 T1: 45 T2: 45 T3: 45	T0: 32 T1: - T2: 8 T3: 3	T0: 6 T1: 6 T2: 4 T3: 3	T0: 3 T1: 3 T2: 4 T3: 2	T0: 3 T1: 3 T2: 3 T3: 3	T0: 3 T1: 3 T2: 3 T3: 3	Bilateral IACs

^a^Additive and progressive MRI score [40, 41]

^b^Metylprednisolone acetate

*Inj* injection, *Medic* medication, *MIO* maximal incisal opening, *MRI* magnetic resonance imaging, *FU* follow-up, *MTX* methotrexate, *BioCo* biologics alone or in combination with other, *DMARDs* disease modifying antirheumatic drugs, *JIA* juvenile idiopathic arthritis, *TMJ* temporomandibular joint, *IACs* intraarticular corticosteroid injections; T0 = Pre-injection, T1 = 2-months follow-up, T2 = 1-year follow-up, T3 = 2-year follow-up; − missing data " - "

## Discussion

To our knowledge, this is the first prospective study using two recently published MRI scoring systems to assess the efficacy and safety of IACs in the TMJ in adolescents with JIA. We found that a single IAC in combination with systemic therapy may improve short-term and long-term MRI-assessed inflammation and MIO, even though pain and MRI-assessed damage did not improve significantly.

### Clinical outcomes: pain and MIO

In our study the pain-index score improved in 6/11 patients at 2-months follow-up and 8/10 patients at the 2-year follow-up median 22 months after IACs to the TMJs. Pain was one of the main indications for performing the IACs in our study, and the pain-index score is reported to be a valid and sensitive outcome measure in TMJ arthritis [18]. Improvement in pain is reported in most retrospective studies in JIA children based on medical chart information or the patients’ self-assessment of pain, where improvement in orofacial symptoms is seen in 67–100%, follow-up ranging from mean 3 to 52 months after TMJ-IACs [16, 23, 28, 44, 45]. However, none of these studies used quantified pain reports. Stoustrup et al. used the validated pain-index score in 13 JIA children receiving IACs to the TMJs in a prospective pilot study [18]. They found significant short-term pain reduction, but remitting pain at long-term follow-up, indicating a loss of the initial effect of the IACs [18]. Our study shows a trend for improvement in pain at the 2-month follow-up, which is sustained during the observation period over 2 years (not statistically significant). The sustained tendency of reduced pain may be due to the systemic medication (DMARDs and biologics), which was changed in 10/15 patients in our study. Five of the 11 patients were in remission at the 2-year follow-up, indicating an effect of the treatment, which included IACs and the systemic medication. The sampling procedure with lavage may also induce improvement, Olsen-Bergem et al. found that arthrocentesis with lavage in patients with TMJ arthritis and JIA might be beneficial for the treatment outcome, and that steroids did not add additional effect to the outcome [46]. The natural fluctuation with waxing and waning disease activity over time often seen in JIA must also be considered [47].

In line with most studies on treatment efficacy in JIA we did not have improvement in all the cases receiving TMJ IACs, 2/11 did not have improvement in pain and 3/11 had unchanged pain and 3/13 did not have improvement in MIO at 2 months follow-up as detailed in Table 4.

We found that MIO improved in 9 of 13 patients at 2-months follow-up, and in 9 of 11 patients between baseline and 2-year follow-up after IACs to the TMJs. This is similar to retrospective studies where improvements in MIO are reported between 2.7 and 6.6 mm [16–19, 23, 28, 44, 45]. However, measurements of MIO are associated with much variation [48, 49], increase with age, and show a wide normal range in children of the same age [50]. In our study we used standardized protocols and calibration of the examiners in order to avoid measurement bias [35, 36]. Stoustrup et al. found the smallest detectable difference in repeated MIO measurements in patients with JIA to be 5 mm when a strict and standardized measurement protocol with repeated measurements were applied [49]. A clinically relevant improvement ≥5 mm was found in our study in 4 of 13 patients between baseline and 2-months follow-up and in 5 of 11 between baseline and 2-year follow-up after IACs. Our median improvement in MIO may be influenced by random error within the measurement procedure. Moreover, MIO at baseline was not severely reduced, and we doubt that this small change in MIO is a clinically relevant effect on jaw function even if statistically significant.

### MRI outcomes

MRI-verified TMJ-arthritis is not always accompanied by clinical symptoms from the TMJ.

A systematic review concluded that no single clinical finding could accurately predict MRI findings consistent with arthritis [51]. The measurements in our study therefore included both standardized clinical assessment tools with pain reports, MIO, and MRI to verify TMJ-arthritis both at both baseline and follow-up.

A problem in evaluating outcome after IACs has been the use of qualitative assessments and lack of consistent definitions and MRI-scoring systems [40, 41]. In the assessment of inflammation in our study, the additive inflammatory domain improved significantly between baseline and the 2-months follow-up. Improvement was seen in 6/13 patients as compared to 4/13 patients in the progressive inflammation score. At the 2-year follow-up, 7 of the 10 patients improved significantly in the additive inflammatory domain and 3 were stable, whereas in the progressive inflammation score 5 of 11 patients improved at 2-year follow-up, but no overall significant improvement was seen.

The improvement in MRI-assessed inflammation is in accordance with Resnick et al. who found reduced synovial enhancement in their retrospective study of 29 JIA patients with 50 TMJs, even if only 18% of their TMJs experienced complete resolution of synovitis [19]. Most studies of IACs to the TMJs in patients with JIA report an MRI improvement of 48–83% regarding inflammation [16, 17, 27, 28]. However, these studies used different definitions of MRI improvement than the MRI scoring systems used in our study [40, 41].

We found no improvement in the additive damage domain comprising condylar flattening, erosions and disc abnormalities or in the progressive osseous deformity score. Importantly, increased damage was not found at the group level, even if 2 of the 10 patients with 2-year follow-up in our study worsened in the additive damage domain and 1 of 11 patients worsened in the progressive score for osseous deformity. Furthermore, two patients with only 1-year follow-up worsened. We cannot discern the effect of IACs from the effect of ongoing arthritis, even if both patients with 2-year follow-up had repeated IACs once unilaterally. MRI showed persistent inflammation at 2-year follow-up in both these patients. Three patients improved from baseline to 2-year follow-up and one patient improved at 1-year follow-up. Furthermore, another patient who worsened in bone damage between injection and 2-months follow-up, improved at the 2-year follow-up.

Stoll et al. [17] found in 15/47 (32%) of the TMJs injected with IACs, evidence of new-onset erosion and flattening. Arabshahi et al. [16] also reported post-therapeutic progression with bony resorption in three (16%) of 19 TMJs. Ringold et al. [44] reported that 10/15 (67%) of the patients receiving IACs therapy showed signs of worsening. Lochbuhler et al. [25] reported progressive osseous deformation in 45 of 66 TMJs in their cohort of children with JIA and TMJ arthritis receiving repeated injections (mean 2.4 ± 1.4 IACs per joint, range 0–7).

The additive inflammatory MRI score consists of five domains. The fact that this additive inflammatory scoring system have scores 0 to 2 (maximum 2) for joint effusion, joint enhancement and synovial thickening, while bone marrow edema, bone marrow enhancement scores maximum 1, place less emphasis on the two latter domains. The same applies to the additive damage MRI score consisting of three domains, where condylar flattening and erosions score 0 to 2, and disc abnormalities score maximum 1 if present. It is also unclear how the progressive system was constructed with regard to relative weighting of findings. Whether this emphasis is based on data analyses or constructed for simplicity is not stated in the publications of the scores.

The progressive inflammation and osseous deformity scores incorporate several features into one score in a progressive manner. In one of the four papers [40] where the system has been presented it is stated that the most severe change is the deciding feature, however, in the three others [25, 41, 42] this statement is not included. Deciding the most severe change can be challenging, and our interpretation of the system was that a given score was reliant on fulfillment of the previous level of pathology. This may have lowered the progressive osseous deformity score if erosions were present without co-occurrence of flattening, and the progressive inflammation score if bone marrow oedema was present without increased synovial enhancement. There was some variation in the kappa coefficients, both between right and left side in both systems and between the different variables of the additive system. The best intra-observer agreement was found for disc abnormalities and synovial thickening, in the latter with substantial and almost perfect agreement. The repeatability varied somewhat more in our study compared to the report by Tolend et al. [40]. Assessment variations may have influenced the outcome, particularly because of the small study sample. Further assessment of the precision of the published scoring systems including inter-rater repeatability is warranted. The scoring systems have not previously been clinically validated and their ability to detect change has not been examined.

Whether MRI should be performed to assess the effect of interventions in TMJ remains unanswered. In Table 4 there is no uniform pattern, but a trend that the MRI changes in inflammatory scores parallel the clinical improvement. Based on our data, clinical experience and the literature, repeated MRI might be indicated primarily if clinical symptoms and signs do not improve.

### TMJ Arthritis and anterior disc displacement (ADD)

Adolescents with anterior disc displacement (ADD) may have similar inflammatory changes in the TMJ as adolescents with JIA. According to Kellenberger et al., TMJs with ADD show a better-preserved and often normal shape of the glenoid fossa [52]. Even if ADD is not common in JIA it may occur, representing a differential diagnostic challenge with regard to rheumatic or non-rheumatic disease [53].

### IACs techniques

In this study the IACs were performed by arthrocentesis with steroids with the Alstergren push-and-pull technique [26] without imaging guidance. This push-and-pull technique, using a solution of vitamin B12 and physiological salt water, allows sampling of even very little amount of fluid in the TMJ for microbiology and immunology assessments together with the local steroid intervention.

Triamcinolone hexacetonide was mostly used in our study and is shown to be superior to other corticosteroids in a randomized controlled trial (RCT) in large joints [54], only one patient received metylprednisolone acetate [30]. Even if IACs to the TMJs can be safely performed without radiologic guidance [17, 19], different imaging guiding techniques are available. Parra and coworkers showed intraarticular position of the ultrasound (US) - guided needle, confirmed with a CT -scan in 91% of 127 TMJ injections [23]. In accordance with our clinical experience, other studies found ultrasound less sensitive for detecting TMJ synovitis and less feasible to guide TMJ injections [27, 55]. The use of CT-radiation should be minimized in growing children. Fritz et al. concluded that real-time MRI-guided selective injection procedures of the TMJs are feasible, accurate, and safe when performed on a clinical open-bore 1.5-T MR system [22]. However, special non-magnetic needles must be used, MRI-guiding is a logistic challenge, time-consuming and a limited resource in clinical practice [19, 22].

### Side effects

In our study no severe side effects occurred in terms of infection, bleeding or intraarticular calcifications, even if a computed tomography (CT) scan may better show calcifications. However, 2 patients had worsening in bone damage at 2-year follow-up. In addition two patients had worsened score for bone damage at 1-year follow-up but have not yet reached 2-year follow-up. However, these patients had all ongoing inflammation and one of them a trauma to the mandible that may explain the damage. Another patient worsened in bone damage between injection and 2-months follow-up but improved at the 2-year follow-up. Furthermore, three patients improved in bone damage from baseline to 2-year follow-up and 1 patient improved at 1-year follow-up.

Other studies have reported short-term adverse effects such as facial swelling, skin atrophy, pain, TMJ stiffness, chewing dysfunction, fever and TMJ calcifications/ossifications [16, 17, 23, 44, 45, 56]. A chart review by Ringold et al. [56] described heterotopic ossification in the TMJ in children receiving 1–5 TMJ IACs, but the authors were unable to say whether these ossifications were the result of the IACs treatment or due to severe, long-standing TMJ inflammation. Also Lochbuhler et al. [25] reported severe side effects such as ossifications in the TMJs after repeated IACs. Rate of osseous deformities increased from 51% at baseline to 62% at the end of their study, with progression to severe condylar destruction in 26% of joints including 24% with development of intraarticular calcifications / ossifications. Importantly, mandibular growth rate was reduced compared to the normal age- and sex-matched mean growth rate. In that study injections were however performed repeatedly. It is unclear whether the adverse effect of ossifications and reduced mandibular growth is a problem mainly of repeated steroid injections. We could not evaluate the effects on mandibular growth since the patients were mostly fully grown at the time the injection was performed. Even if systemic treatment alone seems to improve TMJ arthritis in most children with JIA in a retrospective study [15], our prospective pilot study may point to a single steroid injection as a treatment option for severe symptoms of TMJ-arthritis unresponsive to systemic treatment in skeletally mature individuals.

### Study strengths and limitations

A strength of the present study is the prospective study design with standardized examination and MRI protocols in a clinical setting. MRI scoring assessments were performed by two experienced specialists and masked regarding whether the images were pre- or post-treatment. In addition, the clinical examiners used standardized examination protocols and were repeatedly calibrated, even if recalibration not necessary always change the inter-examiner reliability [37]. Our study sample is comparable to population-based JIA cohorts and case-control studies regarding gender and JIA category distribution [7, 8, 17]. A limitation is that clinical examiners and the patients were not masked before and after treatment, when assessing clinical variables such as MIO and pain. It must be emphasized that the patient group is small, and therefore the statistical analyses of the main clinical and imaging outcomes, and the discrepancies between the two scoring systems must be interpreted with caution. We found considerable intra-observer variability for some domains of the MRI scores, we did not assess inter-observer variability, and our interpretation of the scoring systems may differ from that of the original authors.

## Conclusion

We found that a single IAC in JIA-patients with TMJ arthritis may reduce MRI-assessed inflammation, and improve mouth-opening capacity minimally. At the 2-months follow-up the pain-index score had improved in 6/11 patients but the change in pain-index score did not reach significance. Condylar bone damage was mostly stable but worsened during 2-year follow-up in two patients with repeated IACs and improved in 3 patients but no overall significant improvement was seen. There were no severe side effects. This is the first prospective clinical study using two recently published MRI scoring systems. However, further and larger studies are needed to verify our findings, elaborate on the clinical validity and adjust the scoring systems where needed. The combined effects of naturally fluctuating JIA disease activity, systemic medication changes, and TMJ lavage versus IACs must be considered in the assessment of the present findings. Further prospective clinical studies on adolescents with an age not critical for mandibular growth retardation due to steroid injection, including a control group, are needed in order to fully elucidate the effect of IACs on TMJ arthritis.

## Supplementary information

**Additional file 1: Supplementary Table 1.** Magnetic resonance imaging (MRI) protocol for adolescents with juvenile idiopathic arthrtitis (JIA) and temporomandibular joint (TMJ) arthritis receiving intraarticular corticosteroids (IACs).

## Data Availability

Data can be obtained from the corresponding author, Paula Frid: paula.frid@unn.no

## Abbreviations

ACR: American College of Rheumatology
Biologic comb: Current use of Biologics alone or in combination with MTX
CRP: C-reactive protein
CT: Computed tomography
DC/TMD: Diagnostic criteria for temporomandibular dysfunction
DMARDs: Disease-modifying anti-rheumatic drugs
ESR: Erythrocyte sedimentation rate
IACs: Intraarticular corticosteroids
ILAR: International League of Associations for Rheumatology
JADAS: The composite juvenile arthritis10-joint disease activity score
JIA: Juvenile idiopathic arthritis
MDgloVAS: Physician global evaluation of overall disease activity on a 10-cm visual analogue scale
MIO: Maximal incisal opening
MRI: Magnetic resonance imaging
MTX: Methotreaxthe
NorJIA: The Norwegian JIA Study
NSAIDs: Non-steroidal anti-inflammatory drugs
PAS: Posterior airway space
PRgloVAS: Patient reported global assesment of well-being
PRpainVAS: Patient reported pain VAS
RF: Rheumatoid factor
SD: Standard deviations
TMJ: Temporomandibular joint
US: Ultrasound
VAS: Visual analogue scale
