# Supplementary material for: Efficacy and safety of intraarticular corticosteroid injections in adolescents with juvenile idiopathic arthritis in the temporomandibular joint: a Norwegian 2-year prospective multicenter pilot study
Source: Pediatr Rheumatol Online J. 2020 Oct 1;18:75. doi: 10.1186/s12969-020-00464-3 (PMC7528594; doi:10.1186/s12969-020-00464-3)
Supplement: Supplementary file 1 — Additional file 1: Supplementary Table 1. Magnetic resonance imaging (MRI) protocol for adolescents with juvenile idiopathic arthrtitis (JIA) and temporomandibular joint (TMJ) arthritis receiving intraarticular corticosteroids (IACs). [file 12969_2020_464_MOESM1_ESM.docx]

**Supplementary Table 1.**

| **Protocol A - 3.0 Tesla** | | | | | | | |
| --- | --- | --- | --- | --- | --- | --- | --- |
| n (total=50) | 49 | 49 | 49 | 49 | 48 | 46 | 2 |
| Weighting | Proton density | T2 | T1 | T1 | T1 | Proton density | T1 |
| Scanning sequence | turbo spin echo | turbo spin echo | turbo spin echo | turbo spin echo | turbo spin echo | turbo spin echo | turbo spin echo |
| Plane | oblique sagittal | oblique sagittal | coronal | oblique sagittal | oblique sagittal | oblique sagittal | oblique sagittal |
| Fat saturation | - | yes | - | yes | yes | - | yes |
| Post i.v. contrast agent (delay) | - | - | - | - | yes (4 min) | yes | yes |
| TE (ms) | 22-43 | 71 | 7.9 | 7.9-8.1 | 7.9-8.1 | 22-43 | 7.9-8.1 |
| TR (ms) | 3470 | 3470-3530 | 700 | 604-758 | 604-726 | 3470 | 604-726 |
| Flip angle (degrees) | 150 | 150 | 131 | 131 | 131 | 150 | 131 |
| Field of View (mm*mm) | 150*150 | 150*150 | 179*179 | 179*179 | 179*179 | 150*150 | 150*150 |
| Acquisition matrix | 314*448 | 314*448 | 359*448 | 359*448 | 359*448 | 314*448 | 269*384 |
| Slice thickness (mm) | 2 | 2 | 2 | 2 | 2 | 2 | 2 |
| Slice spacing (mm) | 2.2 | 2.2 | 2.2 | 2.2 | 2.2 | 2.2 | 2.2 |
| Number of signal averages | 1 | 2 | 3 | 3 | 3 | 1 | 3 |
| Echo train length | 10 | 9 | 4 | 4 | 4 | 10 | 4 |
| Bandwidth (Hz/pixel) | 235 | 230 | 415 | 415 | 415 | 235 | 415 |
| Open mouth position | - | - | - | - | - | yes | yes |
| **Protocol B - 1.5 Tesla** | | | | | | | |
| n (total=7) | 7 | 7 | 7 | 7 | 7 | 7 |  |
| Weighting | T2 | T2 Dixon | T1 | T1 | T1 | T1 |  |
| Scanning sequence | turbo spin echo | turbo spin echo | turbo spin echo | turbo spin echo | turbo spin echo | gradient |  |
| Plane | oblique sagittal | transverse | oblique sagittal | oblique sagittal | oblique coronal | oblique sagittal |  |
| Fat saturation | yes | yes | - | yes | yes | yes? |  |
| Post i.v. contrast agent (delay) | - | - | - | yes (0 min) | yes | yes |  |
| TE (ms) | 80 | 102 | 9.2 | 9.2 | 8.9 | 9.84 |  |
| TR (ms) | 2240 | 2340 | 386 | 386 | 425 | 178 |  |
| Flip angle (degrees) | 150 | 150 | 150 | 150 | 150 | 30 |  |
| Field of View (mm*mm) | 140*140 | 140*140 | 140*140 | 140*140 | 140*140 | 140*140 |  |
| Aquisition matrix | 256*256 | 256*256 | 256*256 | 256*256 | 192*192 | 320*320 |  |
| Slice thickness (mm) | 2 | 2 | 2 | 2 | 2 | 2 |  |
| Slice spacing (mm) | 2 | 2 | 2 | 2 | 2 | 2 |  |
| Number of signal averages | 2 | 2 | 3 | 3 | 2 | 1 |  |
| Echo train length | 16 | 17 | 3 | 3 | 3 | 1 |  |
| Bandwidth (Hz/pixel) | 200 | 245 | 200 | 200 | 200 | 70 |  |
| Open mouth position | - | - | - | - | - | open |  |
